# Serum Creatinine Level: A Supplemental Index to Distinguish Duchenne Muscular Dystrophy from Becker Muscular Dystrophy

**DOI:** 10.1155/2015/141856

**Published:** 2015-03-17

**Authors:** Huili Zhang, Yuling Zhu, Yiming Sun, Yingyin Liang, Yaqin Li, Yu Zhang, Langhui Deng, Xingxuan Wen, Cheng Zhang

**Affiliations:** ^1^Department of Neurology, First Affiliated Hospital, Sun Yat-sen University, No. 58 Zhongshan 2nd Road, Guangzhou 510080, China; ^2^Department of Anatomy and Neurobiology, Zhongshan School of Medicine, Sun Yat-sen University, No. 58 Zhongshan 2nd Road, Guangzhou 510080, China; ^3^Department of Healthcare Clinic, First Affiliated Hospital, Sun Yat-sen University, No. 58 Zhongshan 2nd Road, Guangzhou 510080, China; ^4^Department of Laboratory Medicine, First Affiliated Hospital, Sun Yat-sen University, No. 58 Zhongshan 2nd Road, Guangzhou 510080, China; ^5^Department of Epidemiology and Health Statistics, School of Public Health, Sun Yat-sen University, No. 58 Zhongshan 2nd Road, Guangzhou 510080, China

## Abstract

*Background.* To improve assessment of dystrophinopathy, the aim of this study was to identify whether serum creatinine (Crn) level reflects disease severity. *Methods.* Biochemical, Vignos score, and genetic data were collected on 212 boys with dystrophinopathy. *Results.* Serum Crn level had a strong inverse correlation with Vignos score by simple correlation (*r* = −0.793) and partial correlation analysis after adjustment for age, height, and weight (*r* = −0.791; both *P* < 0.01). Serum Crn level was significantly higher in patients with in-frame than out-of-frame mutations (*Z* = −4.716,  *P* < 0.01) and in Becker muscular dystrophy (BMD) patients than Duchenne muscular dystrophy (DMD) patients at ages 4, 5, 7, and 9 yr (all *P* < 0.0125). After adjusting for age, height, and weight, BMD patients still had a significantly higher serum Crn level than DMD patients (*β* = 7.140,  *t* = 6.277,  *P* < 0.01). *Conclusions.* Serum Crn level reflected disease severity and may serve as a supplemental index to distinguish DMD from BMD in clinical practice.

## 1. Introduction

Duchenne muscular dystrophy (DMD; MIM number 310200) and Becker muscular dystrophy (BMD; MIM number 300376) are allelic dystrophinopathies caused by mutations in the dystrophin gene (MIM number 300377) [1]. It is widely accepted that out-of-frame mutations in the dystrophin gene lead to DMD, whereas in-frame mutations result in BMD; this is known as the “reading-frame rule” [2]. DMD is a more severe phenotype, and most patients become confined to a wheelchair by the age of 12, whereas BMD is a milder phenotype with near-normal life expectancy [3]. Multiple therapeutic approaches are being explored in preclinical and clinical trials, such as restoring dystrophin expression, preventing the downstream effects of muscle degeneration, and promoting muscle growth or replacement [4]. Consequently, responsive outcome measures are needed to quantify the disease severity of dystrophinopathy patients.

Determination of muscle dystrophin protein expression with immunohistochemistry and/or immunoblotting has been a reliable assay to monitor disease progression. However, due to its inherent invasiveness, it is not realistic to perform muscle biopsy frequently to monitor response to therapy. A battery of clinical outcomes have subsequently been used to determine changes, including quantitative muscle strength tests using dynamometry [5], motor function scales [6, 7], and timed function tests [8]. However, these are difficult to apply in very young children, are time-consuming, and are less objective, being more likely to be influenced by a rater's experience. Recently, several studies have illuminated the role of microRNAs as serum biomarkers for monitoring disease severity in DMD patients [9, 10], although studies on a larger number of patients are needed to validate this. Accordingly, reliable, valid, and convenient assessment of disease progression is a challenge in clinical practice.

Creatinine (Crn), an end product from the nonenzymatic cyclization of creatine and phosphocreatine, is formed almost exclusively in skeletal muscle. It has been demonstrated that Crn excretion progressively decreases in parallel with muscle wasting in patients with DMD [11], and 24-hour urinary Crn excretion may be a reliable index of muscle mass [12]. Considering that serum Crn correlated strongly with urinary Crn excretion [13] and that the more severe DMD patients appear to have a lower serum Crn level than the milder BMD patients, we suggested that serum Crn level might serve as a useful index to assess disease severity. However, this has not yet been formally documented.

In this study, we present clinical, biochemical, and motor function score and genetic data on a large cohort of Chinese patients with dystrophinopathy and exhaustively assess the relationship between serum Crn level and disease severity. Monitoring serum Crn level would be an easier and more convenient method to assess disease progression, especially for very young patients.

## 2. Patients and Methods

### 2.1. Patients

Two hundred and twelve boys (mean age, 10.8 ± 5.4 yr) with dystrophinopathy who attended the Neuromuscular Clinic at the First Affiliated Hospital of Sun Yat-sen University for regular visits participated in this study. The diagnosis of dystrophinopathy was based on clinical, biochemical, and molecular analysis or immunohistochemistry and Western blotting for dystrophin in a muscle biopsy specimen. The clinical phenotype (DMD versus BMD) was defined mainly by clinical presentation. For children aged <5 yr, those presenting with no weakness and demonstrating residual dystrophin expression via immunohistochemistry were categorized as BMD. Patients were excluded from the study if they had any coexisting medical diseases or had a family history of renal disease. The study was approved by the Local Ethical Committee of Sun Yat-sen University, and informed consent was obtained from the parents or older patients (age ≥ 18 yr).

### 2.2. Laboratory Measurements

A 4 mL blood sample was drawn from the peripheral vein of patients after a 10-hour fast. Twelve patients (age ≤ 1 yr) and four patients (age 2-3 yr) did not have blood drawn, and an additional six patients (age > 10 yr) did not receive a blood test because the parent did not provide permission. In total, 190 patients underwent the blood test. No subject was on a meat-free diet. Serum Crn was measured in the hospital's laboratory department. Serum Crn (normal value > 53 *μ*mol/L) was assayed via enzymatic reaction as per the kit instructions (Abbott Aeroset Fully Automatic Biochemical Analyzer, Abbott Laboratories, USA). Serum cystatin C level was also measured to exclude renal disease [14]. Creatine kinase and routine biochemical analysis was also conducted simultaneously.

### 2.3. Height Measurement

Five patients (two ambulant and three nonambulant) with prominent scoliosis and three patients (nonambulant) withlimb contractures were excluded. For disabled patients (e.g., wheelchair-dependent patients or patients with ankle joint contracture), body height was measured twice with the patient lying supine by different investigators. The mean height value was used.

### 2.4. Evaluation of Motor Function

From June 2012 to October 2014 each patient (age > 1 yr) was evaluated once by the same physiotherapist using the Vignos scale [6]. For children <3 yr of age and those not cooperative for evaluation, the physiotherapist evaluated them by indirect observation in the clinic and based on information from the parents. The Vignos scale is a 10-level classification for evaluating functional activity of the lower limbs, with grades ranging from 1 to 10: 1 indicates the patient is able to walk and climb stairs without assistance, while 10 indicates the patient is bedbound.

### 2.5. Genetic Analysis

Genomic DNA was extracted from peripheral blood using a DNA extraction kit as per the manufacturer's instructions (Qiagen, Hilden, Germany). Multiplex ligation-dependent probe amplification reactions were performed to detect exon deletions and duplications. Denaturing high performance liquid chromatography reactions were performed to detect other mutations.

Using the Leiden DMD gene reading-frame checker (http://www.dmd.nl/index.html) [15], we examined cases with large rearrangement mutations (deletions or duplications) to determine if the changes resulted in either an out-of-frame or in-frame mutation.

### 2.6. Statistical Analysis

Statistical analyses were performed using SPSS, version 20.0 (IBM SPSS Statistics 20.0). Normal distribution of the variables was tested with the Kolmogorov-Smirnov test (*n* > 50) or Shapiro-Wilk test (*n* ≤ 50). Variables (distributed normally) were reported as mean and standard deviation (mean ± SD) and otherwise as median and the 25th and 75th percentile (M (*P*_25_–*P*_75_)). Correlation between serum Crn level and Vignos scale score was evaluated using the Spearman correlation test and reevaluated using the partial correlation test after adjusting for age, height, and weight. Serum Crn level was compared with out-of-frame and in-frame mutations using the Mann-Whitney rank-sum test. Serum Crn level was compared between DMD and BMD patients using the Mann-Whitney rank-sum test, Bonferroni test, and multiple linear regression analysis (adjusting for age, height, and weight). All tests were two-tailed, and *P* values <0.05 were considered statistically significant.

## 3. Results

### 3.1. Clinical Data

Among the 212 patients analyzed in this study, 148 had DMD, 52 had BMD, and twelve had intermediate muscular dystrophy (IMD). As the 12 patients were <1 yr of age, it was not possible to distinguish between DMD and BMD. Consequently, it was categorized as IMD. The median age for DMD was 7 yr (5–9 yr), whereas the median age for BMD was 9 yr (5–17 yr). Of the 190 patients who underwent a blood test, all had a normal serum cystatin C level (data not shown) and an abnormal serum Crn level, which was remarkably decreased at a median value of 17.00 *μ*mol/L (14.00–21.25 *μ*mol/L). Of the 195 patients who underwent the gene test, large rearrangements were identified in 136 patients (69.74%), and smaller mutations were identified in 42 patients (21.54%). In the remaining 17 patients, large rearrangements were not detected, but tests for smaller mutations were not further performed (Table 1).

### 3.2. Serum Crn Level Correlated Inversely with Vignos Scale

**Figure 1:**
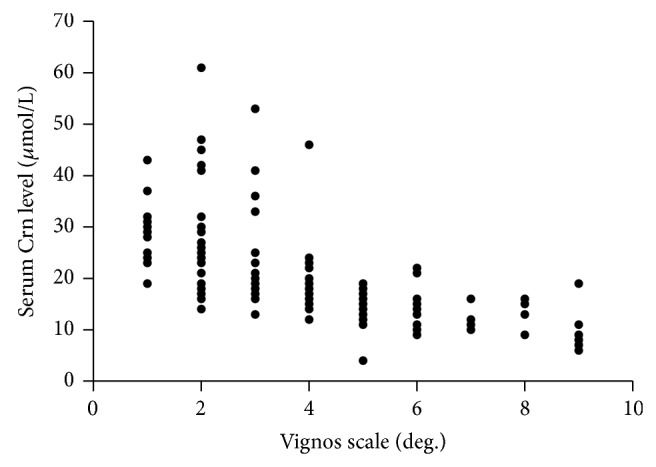
Serum creatinine (Crn) level is inversely correlated with Vignos scale scores.

Vignos scale data were available for only 187 patients. Serum Crn level had a strong inverse correlation with Vignos score (*r* = −0.793, *P* < 0.05; Figure 1); this correlation remained after adjustment for age, height, and weight (*r* = −0.791, *P* < 0.05).

### 3.3. Serum Crn Level Differed in Patients with Out-of-Frame versus In-Frame Mutation

**Figure 2:**
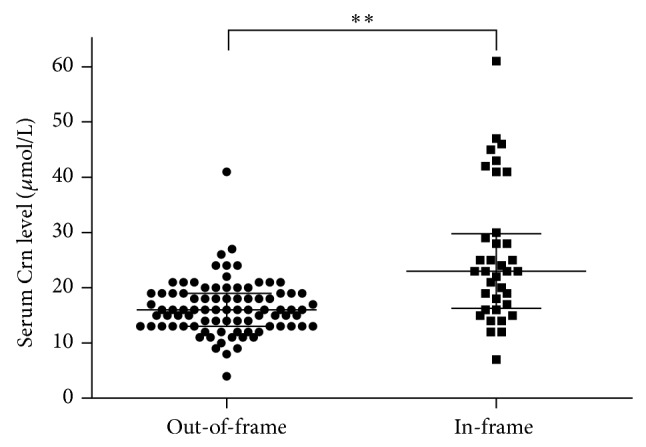
Serum creatinine (Crn) level differed in patients with out-of-frame versus in-frame mutation (^**^ *P* < 0.01).

Of the 136 patients with a large rearrangements mutation, 94 (69.12%) had an out-of-frame mutation and 42 (30.89%) had an in-frame mutation. Median serum Crn level was significantly higher in patients with in-frame mutation (23.00 *μ*mol/L [16.25–29.75]) than those with out-of-frame mutation (16.00 *μ*mol/L [13.00–19.00]; *Z* = −4.716, *P* < 0.05; Figure 2).

### 3.4. Serum Crn Level Differed in DMD Patients and BMD Patients

Median serum Crn level (16.00 *μ*mol/L [13.00–18.00]) was significantly higher in BMD patients than in DMD patients (26.50 *μ*mol/L [23.00–37.00]; *Z* = −9.068, *P* < 0.01). Since age in DMD patients was different than BMD patients (*P* < 0.05), we made further comparisons based on 1-year age intervals for patients. Patients aged 2–12 yr were chosen because patients <1 yr of age cannot be diagnosed with DMD or BMD, and few DMD patients >12 yr of age visited regularly. BMD patients had a significantly higher serum Crn level than DMD patients at ages 4, 5, 7, and 9 yr (Table 2). Although statistical analysis could not be performed for ages 3, 6, 11, and 12 due to the small sample size in BMD patients, serum Crn level tended to be higher in BMD patients than in DMD patients. After adjustment for age, height, and weight, BMD patients still had a significantly higher serum Crn level than DMD patients (*β* = 7.140, *t* = 6.277, *P* < 0.01).

## 4. Discussion

This study shows that serum Crn level is significantly related to measures of disease severity, including functional grading scores, genetic data regarding reading-frame mutations, and clinical phenotype (DMD versus BMD).

To corroborate that serum Crn level may be a biomarker for assessing severity of dystrophinopathy, we first used the Vignos scale to attain finer gradations in disease progression. The Vignos scale has been shown to have good inter- and intrarater reliability [16] and has been extensively adapted to monitor disease progression and assess the response to treatment [17, 18]. Moderate to strong inverse correlations between serum Crn level and the Vignos scale were detected; this suggests that patients with a lower serum Crn level may present a more severe disease state with more seriously impaired motor function.

Furthermore, given that the Vignos scale was more subjective, we evaluated disease severity by genetic analysis. Patients with in-frame mutations (refers to a milder phenotype) had significantly higher serum Crn levels than those with out-of-frame mutations (refers to a more severe phenotype), suggesting that higher serum Crn level indicates a milder disease status.

However, the reading-frame rule held true for 86.4% of the DMD patients and 74.55% of the BMD patients, according to our previous report [19]. Hence, we compared serum Crn level between DMD and BMD patients directly and found that BMD patients had a significantly higher serum Crn level than DMD patients. Additionally, since age influences serum Crn level in normal children [20], we reassessed serum Crn levels between DMD and BMD patients subgrouped by age and similar results were found in patients with some specific age. Nevertheless, a broader interpretation of the results is limited by the low number of BMD patients at younger ages. Finally, after adjustment for age, height, and weight, we still found BMD patients had a significantly higher serum Crn level than DMD patients. All of this further suggests that serum Crn level reflected disease severity.

Several limitations of our study are worth noting. First, the Vignos scale is related primarily to gross motor function. It is practical but not sensitive for assessment of lower limb function and does not assess upper limb function. Therefore, other more comprehensive function scales such as the Motor Function Measure [7] scale are needed. Second, we performed a cross-sectional study to establish a negative correlation between serum Crn level and disease severity. However, a longitudinal study may help to unravel potential mechanistic links between them. For example, we did not monitor serum Crn level in patients with steroid therapy; future studies should validate whether serum Crn level can be used for follow-up of dystrophinopathy patients. Third, we did not thoroughly investigate the effects of some medications on serum Crn level. Some medications interfere with Crn secretion or the Crn assay per se (e.g., administration of certain cephalosporins). In addition, serum Crn level may be altered for patients not in a steady state of Crn balance, such as patients in starvation.

In conclusion, these results support the hypothesis that serum Crn levels are associated with clinical severity and may be a simple biomarker for evaluating disease severity. In addition to muscle biopsy and gene analysis, serum Crn level may be a supplemental index to help clinically distinguish DMD from BMD.

## Figures and Tables

**Table 1:** The number (percentage) of patients available for clinical analysis.

Clinical analysis	Number	Percentage (%)
Clinical phenotype	212	
DMD	148	69.81%
BMD	52	24.53%
IMD	12	5.66%
Blood test	190	
Testing for Crn level	190	100%
Testing for cystatin C level	190	100%
Vignos scale	187	
Degree (1–6)	173	92.51%
Degree (7–9)	14	7.49%
Mutation analysis	195	
Large rearrangements	136	69.74%
Smaller mutations	42	21.54%
No large rearrangements	17	8.72%

DMD: Duchenne muscular dystrophy; BMD: Becker muscular dystrophy; IMD: intermediate muscular dystrophy; Crn: creatinine.

**Table 2:** Serum creatinine levels in DMD and BMD patients (age 2–12 yr).

Age (y)	DMD			BMD			*Z*	*P *
	Patients (*N*)	Mean ± SD/M (*P*_25_–*P*_75_) (*μ*mol/L)	Original value (*μ*mol/L)	Patients (*N*)	Mean ± SD/M (*P*_25_–*P*_75_) (*μ*mol/L)	Original value (*μ*mol/L)		
2	5	15.80 ± 3.962		1		23	N.A.	
3	10	14.95 ± 2.114		2		14; 24	N.A.	
4	13	17.46 ± 4.875		5	26.00 ± 2.236		−2.670	0.008^*^
5	10	15.20 ± 2.530		5	23.60 ± 4.393		−3.009	0.003^*^
6	13	16.69 ± 4.461		2		28; 37	N.A.	
7	16	15.19 ± 2.762		5	25 (24.50–26.50)		−3.321	0.001^*^
8	32	15 (12.25–19.75)		1		21	N.A.	
9	15	14.47 ± 3.623		4	25.25 ± 8.098		−2.824	0.005^*^
10	4	19.75 ± 4.031		0	N.A.	N.A.	N.A.	
11	7	15.14 ± 3.532		2		32; 41	N.A.	
12	3		16; 16; 19	2		18; 31	N.A.	

DMD: Duchenne muscular dystrophy; BMD: Becker muscular dystrophy; SD; standard deviation; N.A.: not available.

Serum creatinine values are reported as mean ± SD (normal distribution) or as median and the 25th and 75th percentiles (M (*P*_25_–*P*_75_)). When *N* < 3, the original serum creatinine value is presented.

^*^ *P* values < 0.0125 are considered statistically significant for multiple comparison by using Bonferroni test.
